# A prospective study on radiation doses to organs at risk (OARs) during intensity-modulated radiotherapy for nasopharyngeal carcinoma patients

**DOI:** 10.18632/oncotarget.7826

**Published:** 2016-03-01

**Authors:** Ji-Jin Yao, Fo-Ping Chen, Guan-Qun Zhou, Wang-Jian Zhang, Lin Xu, Xiao-Ju Wang, Li Lin, Jun Ma, Ying Sun

**Affiliations:** ^1^ Department of Radiation Oncology, Sun Yat-sen University Cancer Center, State Key Laboratory of Oncology in South China, Collaborative Innovation Center for Cancer Medicine, Guangzhou 510060, Guangdong Province, People's Republic of China; ^2^ Department of Medical Statistics and Epidemiology & Health Information Research Center & Guangdong Key Laboratory of Medicine, School of Public Health, Sun Yat-sen University, Guangzhou 510080, Guangdong Province, People's Republic of China; ^3^ Department of Oncology, Guangzhou First People's Hospital, Guangzhou Medical University, Guangzhou 510180, Guangdong Province, People's Republic of China

**Keywords:** nasopharyngeal carcinoma, intensity-modulated radiotherapy, organs at risk, radiation dose, gross tumor volume

## Abstract

This study is to investigate the dose distribution of organs at risk (OARs) in cases of nasopharyngeal carcinoma (NPC). From July 2013 to October 2014, a prospective cohort study involving 148 patients was carried out at our center. OARs surrounding the nasopharynx were contoured on axial CT planning images in all patients. Dose-volume histograms of OARs and gross tumor volumes (GTV) were calculated. Multivariate analysis showed that radiation dose to OARs was associated with T stage and, especially, GTV. Seven OARs, including the spinal cord, eye and mandible, easily tolerated radiation doses in all patients; six OARs including the brain stem, chiasm and temporal lobe easily tolerated radiation doses in patients with a small GTV, but with difficulty when GTV was large; and other nine OARs including the parotid gland, cochlea and tympanic cavity met tolerance doses with difficulty in all patients. According to the patterns of radiation doses to OARs, it may help us to further reduce subsequent complications by improving the efficiency of plan optimization and evaluation.

## INTRODUCTION

Nasopharyngeal carcinoma (NPC) is a malignant disease endemic in Southern China [1]. Due to the anatomical location and radio-sensitivity of non-disseminated NPC, the primary treatment modality is radical radiotherapy. Compared with conventional two-dimensional radiotherapy, intensity-modulated radiotherapy (IMRT) has become the technique of choice since it provides excellent locoregional control and sparing of organs at risk (OARs) in NPC [2, 3].

The frequency of adverse effects, which are usually chronic, irreversible and progressive, is related to the radiation dose to OARs [4]. Although OARs sparing has improved significantly with IMRT, late toxicities such as grade 2 – 4 xerostomia and sensorineural hearing loss still occur in up to 40% of patients [5, 6]. It is well-recognized that total radiation doses and fraction size are associated with the development of radiation toxicities [7]. However, little is known about the radiation dose to OARs surrounding the nasopharynx for NPC patients with different gross tumor volumes (GTVs).

Therefore, we prospectively enrolled 148 NPC patients and investigated the relationship between radiation dose to the OARs and GTV in order to further reduce subsequent complications by improving the efficiency of plan optimization and evaluation.

## RESULTS

### Classification based on GTV

**Figure 1:**
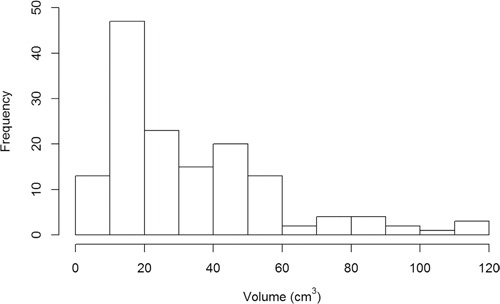
The distribution of patients in each group determined by GTV

GTV was collected for all patients, and median GTV was 25.7 cm^3^ (1.3 – 115.7 cm^3^). According to the distribution of patients determined by GTV (Figure 1), most of GTVs were distributed between 0 – 60 cm^3^, while a small number of GTVs were greater than 60 cm^3^. In order to investigate the relationship between dosimetry variability of OARs and GTV, patients were divided into four groups based on GTV: Group 1 (GTV < 20 cm^3^, n = 59); Group 2 (20 ≤ GTV < 40 cm^3^, n = 39); Group 3 (40 ≤ GTV < 60 cm^3^, n = 34); Group 4 (GTV > 60 cm^3^, n = 16). Radiation doses in different GTVs were shown in Table 1.

**Table 1:** Mean (± SD) of doses to OARs based on GTV for the 148 patients

Organ	Dose volume metrics	Group 1	Group 2	Group 3	Group 4
**BrainStem_PRV**	D1 (Gy)^¶^	53.31±3.41	58.29±5.65	62.20±5.16	68.13±4.74
**SpinalCord_PRV**	D1 (Gy)	38.78±2.24	39.26±2.20	39.46±2.30	40.73±4.99
**OpticNerve_PRV**	D1 (Gy)	33.19±18.49	49.57±13.32	53.52±12.17	60.62±10.75
**Chiasm_PRV**	D1 (Gy)	41.72±12.96	55.81±8.36	60.67±8.90	66.54±8.62
**TemporalLobe_PRV**	D1 (Gy)	55.76±4.93	61.35±5.85	65.65±5.92	70.3±4.74
**Pituitary**	Dmax (Gy)	51.54±8.72	60.93±8.73	64.61±6.88	71.23±5.90
**Submandibular**	Dmean (Gy)	49.94±7.73	51.15±8.09	52.91±7.63	53.88±6.90
**Mandible**	V50 (%)^‡^	12.73±14.33	11.69±13.84	15.77±14.47	23.83±20.32
**TMjoint**	Dmax (Gy)	48.02±9.48	55.99±9.88	57.96±9.02	64.16±10.29
**Lens**	Dmax (Gy)	3.29±1.37	5.48±2.76	7.20±2.58	9.08±3.44
**Eye**	Dmean (Gy)	4.90±3.18	7.86±3.94	9.64±3.24	13.63±6.98
**Parotid**	Dmean (Gy)	33.47±5.91	34.55±5.02	35.84±4.98	37.53±7.15
**Cochlea**	Dmean (Gy)	43.85±5.90	50.02±9.76	54.20±10.20	61.19±9.60
**IAC**	Dmean (Gy)	43.39±4.20	49.63±8.57	54.23±9.59	62.35±9.31
**VestibulSemi**	Dmean (Gy)	37.24±4.25	41.76±7.40	44.93±7.62	51.47±8.21
**Eustachian tube**	Dmean (Gy)	47.69±7.27	52.66±10.04	57.15±9.73	63.02±10.09
**TympanicCavity**	Dmean (Gy)	37.69±5.39	41.49±7.43	43.71±7.75	48.22±9.80
**Mastoid**	Dmean (Gy)	31.82±3.91	34.35±4.21	35.06±4.17	39.39±5.58
**OralCavity**	D1 (Gy)	60.36±4.02	63.31±5.59	65.37±4.95	68.24±5.18
**PharynxConst_S**	Dmean (Gy)	61.25±3.70	63.30±4.70	65.05±4.44	66.04±4.43
**PharynxConst_M**	Dmean (Gy)	56.45±4.73	58.17±5.06	58.57±4.88	59.69±3.18
**PharynxConst_I**	Dmean (Gy)	45.72±2.43	47.95±4.69	47.54±3.93	48.13±2.48

Abbreviations: Group 1, GTV < 20 cm^3^; Group 2, 20 ≤ GTV < 40 cm^3^; Group 3, 40 < GTV < 60 cm^3^; Group 4, GTV > 60 cm^3^. PRV, planing risk volume; TMjoint, temporomandibular joint; IAC, internal auditory canal; VestibulSemi, vestibule and semicircular canal; PharynxConst_S, superior constrictor of pharynx; PharynxConst_M, middle constrictor of pharynx; PharynxConst_I, inferior constrictor of pharynx.

¶ Dose delivered to 1% of the volume.

‡ Percentage volume that received >50 Gy.

The distributions of T stage and N stage in different GTVs are shown in Table 2. There were 16 T1 stage patients, of which 14 patients were in Group 1 and 2 in Group 2. There were no T1 stage patients in either Group 3 or 4. There were 31 T4 stage patients: none in Group 1, 7 in Group 2, 12 in Group 3 and 12 in Group 4. There were 67 N1 stage patients; 21 in Group 1, 22 in Group 2, 16 in Group 3 and 8 in Group 4. Finally, there were 26 N3 stage patients: 9 in Group 1, 7 in Group 2, 6 in group 3 and 4 in Group 4.

**Table 2:** The distribution of T stage and N stage at various volumes of GTV for the 148 patients

	Group 1	Group 2	Group 3	Group 4	Total
**T stage**					
T1 (n, %)	14 (88%)	2 (12%)	0 (0%)	0 (0%)	16 (100%)
T2 (n, %)	25 (78%)	6 (19%)	1 (3%)	0 (0%)	32 (100%)
T3 (n, %)	20 (29%)	24 (35%)	21 (30%)	4 (6%)	69 (100%)
T4 (n, %)	0 (0%)	7 (23%)	12 (39%)	12 (39%)	31 (100%)
**N stage**					
N0 (n, %)	11 (61%)	3 (17%)	4 (22%)	0 (0%)	18 (100%)
N1 (n, %)	21 (31%)	22 (33%)	16 (24%)	8 (12%)	67 (100%)
N2 (n, %)	18 (49%)	7 (19%)	8 (22%)	4 (11%)	37 (100%)
N3 (n, %)	9 (35%)	7 (27%)	6 (23%)	4 (15%)	26 (100%)

Abbreviations: Group 1, GTV < 20 cm^3^; Group 2, 20 ≤ GTV < 40 cm^3^; Group 3, 40 < GTV < 60 cm^3^; Group 4, GTV > 60 cm^3^.

### Radiation doses to PTVs based on GTV

Radiation doses to planning target volumes (PTVs) for four groups based on GTV are summarized in supplementary Table 3. In general, all plans met the planning goals for target coverage, and no patient had a V115 (percentage volume covering 115% of the prescribed dose) of PTV_7000 exceeding 1%. Both V100 (percentage volume covering 100% of the prescribed dose) and V95 (percentage volume covering 95% of the prescribed dose) of PTV_7000, PTV_6000 and PTV_5400 was up to 98-100% in all groups. We did not find any significant dosimetry difference in any groups in terms of PTV_7000, PTV_6000 or PTV_5400 (P > 0.05).

### Variables affecting the radiation doses to OARs

Logistic multifactorial analysis was used to investigate which independent risk factors were associated with radiation dose to OARs (Table 3). Two independent risk factors, GTV and T stage, were significantly associated with the radiation dose to 15 OARs including the brain stem, temporal lobe and cochlea. GTV was found to be a better significant predictor than T stage. For example, the dose to 1% volume (D1) for the temporal lobe was positively correlation with both T stage (OR = 3.79; 95% CI 1.31 – 7.26; P = 0.003) and GTV (OR = 5.23; 95% CI 1.56 – 12.75; P < 0.001). However there was no significant link to N stage (OR = 0.68; 95% CI 0.20 – 2.33; P = 0.211). The radiation doses to parotid gland, submandibular gland and pharyngeal constrictors were significantly associated with N stage (P < 0.001, P = 0.005 and P< 0.05, respectively). In contrast, radiation dose to the spinal cord was not significantly linked to T stage, N stage or GTV (P = 0.890, 0.515, and 0.216, respectively).

**Table 3:** Multivariate analysis of variables on the radiation dose of OARs

Organs	Variable	OR (95% CI)	*P*-value
**BrainStem_PRV**	GTV	2.81 (1.21-6.26)	<0.001
	T stage	2.15 (1.12-5.13)	0.002
	N stage	1.56 (0.16-6.71)	0.884
**SpinalCord_PRV**	GTV	1.09 (0.41-2.84)	0.890
	T stage	1.49 (0.47-4.58)	0.515
	N stage	1.77 (0.78-3.97)	0.216
**OpticNerve_PRV**	GTV	3.12 (1.42-7.12)	<0.001
	T stage	2.69 (1.23-4.70)	0.003
	N stage	1.49 (0.49-4.62)	0.967
**Chiasm_PRV**	GTV	5.51 (1.61-9.78)	<0.001
	T stage	3.31 (1.14-5.79)	0.042
	N stage	2.68 (0.56-5.06)	0.800
**TemporalLobe_PRV**	GTV	5.23 (1.56-12.75)	<0.001
	T stage	3.79 (1.31-7.26)	0.003
	N stage	0.68 (0.20-2.33)	0.211
**Pituitary**	GTV	4.87 (2.06-11.99)	<0.001
	T stage	2.65 (1.28-5.67)	0.024
	N stage	3.34 (0.79-7.21)	0.708
**Mandible**	GTV	4.13 (1.37-7.23)	<0.001
	T stage	3.56 (0.68-6.23)	0.423
	N stage	3.47 (0.77-5.89)	0.302
**TMjoint**	GTV	2.71 (1.37-4.55)	<0.001
	T stage	3.20 (0.53-6.15)	0.068
	N stage	2.99 (0.29-5.14)	0.894
**Lens**	GTV	6.87 (2.96-16.74)	<0.001
	T stage	4.13 (2.04-8.62)	<0.001
	N stage	1.29 (0.56-2.99)	0.388
**Eye**	GTV	6.46 (1.69-12.24)	0.016
	T stage	5.72 (1.37-9.86)	0.037
	N stage	3.45 (0.53-7.97)	0.515
**Parotid**	GTV	3.21(1.96-5.43)	0.016
	T stage	2.42 (1.26-4.71)	0.003
	N stage	3.79 (2.11-6.38)	<0.001
**Submandibular**	GTV	2.11(0.93-3.23)	0.079
	T stage	1.89 (0.78-2.75)	0.162
	N stage	2.67 (1.13-4.18)	0.005
**Cochlea**	GTV	2.63 (1.26-4.55)	<0.001
	T stage	2.12(1.61-3.74)	0.012
	N stage	1.89 (0.79-4.63)	0.864
**IAC**	GTV	3.14 (1.77-5.26)	<0.001
	T stage	2.37 (1.41-4.71)	0.018
	N stage	1.70 (0.66-4.45)	0.523
**VestibulSemi**	GTV	2.89 (1.65-5.12)	<0.001
	T stage	2.44 (1.17-4.18)	0.034
	N stage	1.93 (0.71-3.69)	0.053
**Eustachian tube**	GTV	2.23 (1.37-4.54)	<0.001
	T stage	2.65 (1.83-3.83)	0.019
	N stage	1.72 (0.85-4.91)	0.325
**TympanicCavity**	GTV	2.35 (1.21-4.67)	<0.001
	T stage	1.76 (1.08-4.39)	0.041
	N stage	1.57 (0.09-3.51)	0.205
**Mastoid**	GTV	2.34 (1.51-4.03)	<0.001
	T stage	1.81 (1.42-4.45)	0.025
	N stage	1.11 (0.52-4.25)	0.048
**OralCavity**	GTV	3.25 (1.67-6.21)	<0.001
	T stage	2.74 (1.23-5.75)	0.001
	N stage	1.26 (0.34-5.06)	0.082
**PharynxConst_S**	GTV	1.64 (0.91-2.17)	0.063
	T stage	1.77 (0.54-2.77)	0.079
	N stage	1.69 (1.21-3.36)	0.021
**PharynxConst_M**	GTV	1.78 (0.61-3.43)	0.135
	T stage	2.12 (0.78-4.16)	0.362
	N stage	1.77 (1.35-3.25)	0.031
**PharynxConst_I**	GTV	1.55 (0.64-2.61)	0.212
	T stage	1.26 (0.79-2.31)	0.315
	N stage	2.39 (1.32-3.79)	<0.001

Abbreviations: OR, odds ratios; CI: confidence interval; Other abbreviations as in Table 1.

### Characteristics and patterns of radiation dose to OARs

To quantify the characteristics of overdose in different GTV, we analyzed the incidence of exceeding tolerance doses. OARs were initially classified into four risk grades depending on excess rate: (Grade 0): excess rate <10%; (Grade 1): 10% ≤ excess rate <50%; (Grade 2): 50% ≤ excess rate <75%; (Grade 3): excess rate ≥75%. The excess rates in 7 OARs (the spinal cord, optic nerve, mandible, TM joint, eye, PharynxConst_I, and oral cavity) were below 10% in almost all patients. The excess rates in the brain stem, chiasm, temporal lobe, pituitary, lens, vestibular apparatus and semicircular canal were below 10% in early stage patients (T1-2/N0-1) but up to 90% in advanced stage patients (T3-4/N2-3). The excess rates of the other 9 OARs (the parotid gland, submandibular gland, cochlea, IAC, Eustachian tube, tympanic cavity, mastoid and PharynxConst_S, PharynxConst_M) were 50% – 90% in most of patients. Based on excess rates at different GTVs, OARs were classed as either 1) easily tolerating radiation doses in all patients; 2) easily tolerating doses in patients with small GTV but with difficulty when GTV was large; 3) meeting tolerance doses with difficulty in all patients (Table 4).

**Table 4:** Incidence rates of exceeding tolerance doses for OARs surrounding nasopharynx

Organ	Dose metrics	Group 1	Group 2	Group 3	Group 4
**Pattern 1**					
SpinalCord_PRV	D1 <50 (Gy)^¶^	0% (−)	0% (−)	0% (−)	6.3% (−)
OpticNerve_PRV	D1 <60 (Gy)	0.8% (−)	19.2% (+)	15.2% (+)	46.9% (+)
Mandible	V50 < 30%^‡^	12.7% (+)	12.8% (+)	21.2% (+)	31.3% (+)
TMjoint	Dmax <70 (Gy)	0.8% (−)	5.1% (−)	12.1% (+)	37.5% (+)
Eye	Dmean <35 (Gy)	0% (−)	0% (−)	0% (−)	3.1% (−)
OralCavity	D1 <70(Gy)	0% (−)	10.3% (+)	15.2% (+)	43.8% (+)
PharynxConst_I	Dmean <50 (Gy)	1.7% (−)	18.8% (+)	21.2% (+)	23.1% (+)
**Pattern 2**					
BrainStem_PRV	D1<60 (Gy)	0% (−)	35.9% (+)	60.6% (++)	93.8% (+++)
Chiasm_PRV	D1 <60 (Gy)	0% (−)	30.8% (+)	51.5% (++)	68.8% (++)
TemporalLobe_PRV	D1 <65 (Gy)	1.7% (−)	19.2% (+)	53.1% (++)	84.4% (+++)
Pituitary	Dmax <60 (Gy)	5.1% (−)	53.8% (++)	75.8% (+++)	93.8% (+++)
Lens	Dmax <0.6 (Gy)	6.8% (−)	35.9% (+)	66.7% (++)	75.0% (+++)
VestibulSemi	Dmean <45 (Gy)	3.4% (−)	26.9% (+)	48.5% (+)	71.9% (++)
**Pattern 3**					
Parotid	Dmean <26 (Gy)	100% (+++)	100% (+++)	100% (+++)	100% (+++)
Submandibular	Dmean <35 (Gy)	100% (+++)	100% (+++)	100% (+++)	100% (+++)
Cochlea	Dmean <45 (Gy)	33.9% (+)	65.4% (++)	75.8% (+++)	96.9% (+++)
IAC	Dmean <45 (Gy)	31.4% (+)	64.1% (++)	84.8% (+++)	96.9% (+++)
Eustachian tube	Dmean <53 (Gy)	62.7% (++)	74.4% (++)	90.9% (+++)	90.6% (+++)
TympanicCavity	Dmean <34 (Gy)	72.9% (++)	84.6% (+++)	87.9% (+++)	100% (+++)
Mastoid	Dmean< 30 (Gy)	63.6% (++)	83.3% (+++)	84.8% (+++)	96.9% (+++)
PharynxConst_S	Dmean <50 (Gy)	100% (+++)	97.4% (+++)	100% (+++)	100% (+++)
PharynxConst_M	Dmean <50 (Gy)	84.7% (+++)	94.9% (+++)	97.0% (+++)	100% (+++)

According to the excess rates, OARs were initially classified into four risk grades: (−): excess rate <10%; (+): 10% ≤ excess rate < 50%; (++):50% ≤ excess rate < 75%; excess rate ≥ 75%.

Pattern 1: easily tolerated radiation doses in all patients; Pattern 2: easily tolerated doses in patients with small GTV but with difficulty when GTV was large; Pattern 3: met tolerance doses with difficulty in all patients.

¶ Dose received by 1% of the volume

‡ Percentage volume that received >50 Gy; Other abbreviations as in Table 1.

## DISCUSSION

**Figure 2:**
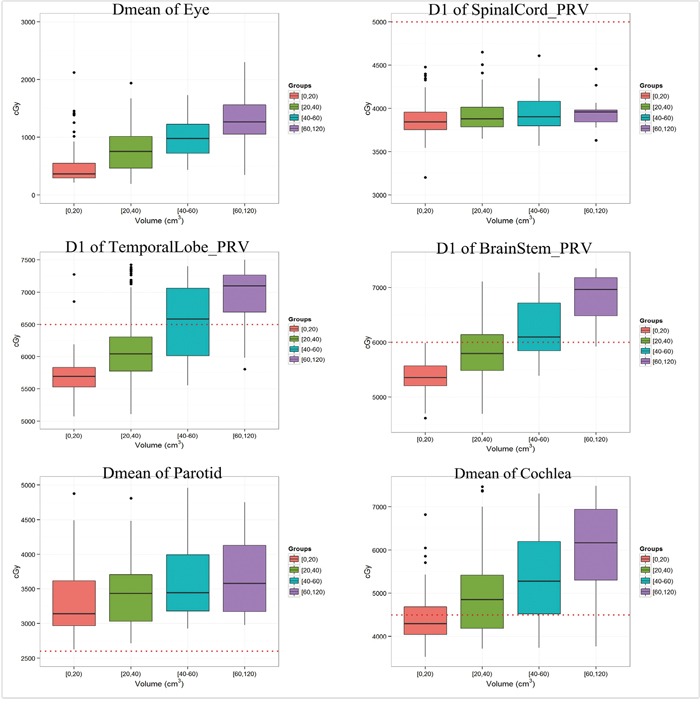
The radiation dose for representative OARs with different GTV; those figures with red-dotted lines refer to tolerance doses for the corresponding OAR

Today, IMRT has been widely used as a more advanced radiation technique for the management of NPC. However, how effective IMRT in sparing OARs around nasopharynx such as temporal lobe, parotid and cochlea is largely unknown. The relationship between GTV and excess rates of OARs was lacking to date. Therefore, we conducted this prospective study in order to directly analyze the dose distribution of OARs in terms of various GTVs in NPC patients treated with IMRT. This study showed that the radiation dose to OARs increased significantly with a larger GTV (Figure 2), and GTV was a useful predictor of radiation dose to OARs around the nasopharynx.

### Radiation doses easily tolerated in most of patients

In most patients, the OARs that were able to tolerate radiation dose easily included the spinal cord, optic nerve, mandible, TM joint, eye, oral cavity and PharynxConst_I. Of these, the eye had the lowest incidence of exceeding the tolerated doses in all groups. We found only case of a patient, in Group 4, whose eye exceeded tolerance doses. For this patient, the maximum dose was 68.86 Gy, and the mean dose was 41.75 Gy (>35 Gy). This patient was found to have a tumor invasion into the orbital apex. Liang et al. [8] reported that the incidence of orbital apex invasion was only 0.1% in NPC patients, which might suggest that the high irradiation dose of certain OARs was dependent on the site of tumor invasion.

Our data also show that the radiation dose to the spinal cord, optic nerve, mandible, TM joint, oral cavity and PharynxConst_I slightly exceeded tolerance doses where GTV was less than 60 cm^3^. In patients where GTV was greater than 60 cm^3^, only 6.3% – 46.9% exceeded tolerance doses. This result suggests that use of IMRT limits radiation exposure to these OARs, especially for patients with a GTV of less than 60 cm^3^. In order to reduce the optimization burden of other OARs, physicists should try to give a relatively lower weighting parameter for those OARs, and clinicians may not have to pay close attention to those OARs in the evaluation of plans.

### Radiation doses were easily tolerated where GTV was small, but with difficulty in larger volumes

For these OARs, the excess rates were very low in patients with a small GTV (< 20 cm^3^), but were over 90% in patients with a large GTV (> 60 cm^3^). Of these OARs, temporal lobe radiation necrosis is a well-recognized and potentially lethal complication of the skull base and central nervous system in patients with NPC [9]. According to QUANTEC-2010 criteria, the incidence of brain radiation necrosis appears to increase as doses exceed 60 Gy in conventional fractionation. In our study, the excess rate in the temporal lobe was only 1.7% in patients with a GTV of less than 20 cm^3^, where it was up to 84.4% in patients with a GTV of over 60 cm^3^. In addition, when tumor volume increased, the D1 of temporal lobe increased to 55.76 ± 4.93 Gy, 61.35 ± 5.85 Gy, 65.65 ± 5.92 Gy and 70.3 ± 4.74 Gy in patients with Group 1, Group 2, Group 3, and Group 4, respectively. As a result, side effects are inevitable. The factors underlying this observation may be interpreted as follows: the large volume of GTV and close proximity to the skull base means that the medial temporal lobes are inevitably included in the target volume.

Our study also showed that the radiation dose to the brain stem, chiasm, pituitary, lens, vestibular apparatus and semicircular canal (VestibulSemi) were less than tolerance doses in most patients with a small GTV (< 20 cm^3^). Therefore, it is suggested that these OARs are of minor concern to clinicians when GTV is less than 20 cm^3^. In addition, the approximate radiation dose received by OARs can be estimated from GTV; if radiation exposure cannot be limited then both clinician and patient must be aware of possible complications.

### Radiation doses were difficultly tolerated in all patients

For this kind of OARs, radiation doses greatly exceeded tolerance levels and the excess rate was up to 100%. It is well recognized that the probability of adverse events is proportional to the radiation dose to the corresponding OARs. The implication is that patients are likely to have a high incidence of related side effects. In a report by Sumitsawan et al. on late complications in NPC patients [10], almost all patients suffered from xerostomia (97.5%). In the present study, we found that the mean doses to the parotid gland and submandibular gland were beyond the tolerance dose in all patients. The factors underlying this observation may be interpreted as follows. First, the parotid gland and submandibular gland were located close to various targets, such as the retropharyngeal lymph node and the site of cervical lymph node drainage. Secondly, to limit the radiation dose to critical structures, such as the brain stem and spinal cord, protection of the parotid gland and submandibular gland was limited. Finally, the parotid gland had a high risk of relapse, and clinicians often define part of the parotid as CTV-2, especially for patients with a large retropharyngeal lymph node and level II lymph node. For these patients, we suggest that clinicians should also contour the normal parotid and submandibular gland (outside of the target areas), and should limit the radiation dose to the normal gland strictly.

Regardless of tumor volume, our study showed that the radiation dose to the auditory apparatus exceeds the tolerance dose in most of patients. Furthermore, radiation dose increased with GTV, up to 70 Gy when GTV exceeded 60 cm^3^. Previous studies demonstrated that high radiation dose (>45 Gy), older age and a high chemotherapy dose (cisplatin) has been found to increase the prevalence of hearing loss, which can impair patient quality of life [11–13]. Pan et al. [14] also reported that almost all cases in which significant hearing loss occurred in the ipsilateral inner ear receiving a high dose of 45 Gy compared with the contralateral ear. With respect to radiation-induced hearing loss, various studies have found the prevalence to be 24% to 57% [10, 15–16]. In order to reduce the incidence of hearing loss and otitis media, it is vital to contour the auditory apparatus (eg. cochlea, eustachian tube, tympanic cavity, and et al.) accurately.

## MATERIALS AND METHODS

### Patient characteristics

Between July 2013 and October 2014, a total of 148 patients with newly diagnosed, biopsy-proven nonmetastatic NPC presented to Sun Yat-sen University Cancer Center and were enrolled in the study. Characteristics of the 148 NPC patients were shown in Table 5. Of the patients, 48 (32.4%) had T1/T2 disease, 100/148 (67.6%) had T3/T4 disease, 85 (57.4%) had N0/N1 disease and 63/148 (42.6%) had N2/N3 disease. The study was approved by the institutional review board (IRB) and all patients provided written informed consent.

**Table 5:** Characteristics of the 148 nasopharyngeal carcinoma patients

Characteristic	No. of patients (%)
**Age (years)**	
Median	42 years
Range	27-76 years
**Sex**	
Male	107 (72.3)
Female	41 (27.7)
**Histology**	
WHO I	1 (0.7)
WHO II/III	147 (99.3)
T category*	
T1	16 (10.8)
T2	32 (21.6)
T3	69 (46.6)
T4	31 (20.9)
N category*	
N0	18 (12.2)
N1	67 (45.3)
N2	37 (25.0)
N3	26 (17.6)
Clinical stage*	
I	4 (2.7)
II	19 (12.8)
III	70 (47.3)
IV	55 (37.2)
**Chemotherapy**	
No	7 (4.7)
Yes	141 (95.3)

* According to the American Joint Committee on Cancer, 7th edition.

### Radiotherapy & chemotherapy

All of the patients received definitive external irradiation. Target volumes were defined using our institutional treatment protocol [17], in accordance with the International Commission on Radiation Units and Measurements reports 50 and 62 [18, 19]. The prescribed dose was 70 Gy to the planning target volume (PTV) of the gross tumor volume (GTV), 64–66 Gy to the PTV of the nodal gross tumor volume (GTV-N), 60 Gy to the PTV of the clinical target volume-1 (CTV-1; high risk regions), and 54 Gy to the PTV of the clinical target volume-2 (CTV-2; low-risk regions) and the nodal regions in the neck (CTV-N) in 33 fractions. The PTVs of GTV, CTV-1, and CTV-2 were termed PTV_7000, PTV_6000, and PTV_5400, respectively. All patients were treated with 1 fraction daily, 5 days a week. Neoadjuvant, concurrent or adjuvant platinum-based chemotherapy was recommended in stage III–IVB NPC.

### Identification of OARs

For the anatomic site specificity of NPC, the irradiation field of traditional radiation therapy usually involved many normal tissues. In the present study, we analyzed the radiation dose to 22 OARs around the nasopharynx including the brainstem, spinal cord, optic nerve and chiasm, the temporal lobe, pituitary, mandible, temporomandibular (TM) joint, lens, eye, parotid gland, submandibular gland, oral cavity, cochlea, internal auditory canal (IAC), VestibulSemi, the Eustachian tube, tympanic cavity, mastoid, superior constrictor of pharynx (PharynxConst_S), middle constrictor of pharynx (PharynxConst_M) and inferior constrictor of pharynx (PharynxConst_I). The Planning Organ-at-Risk Volume of the brainstem and spinal cord was defined as the volume of these organs plus a 3 mm margin, and were termed “BrainStem_PRV” and “SpinalCord_PRV” respectively. Based on anatomic definitions, an experienced radiation oncologist manually contoured OARs on the planning CT scans of the 148 patients with a widely used contouring method [20].

### Plan evaluation of target and OARs

Nine coplanar fields of 6-MV photon beams from a truebeam linear accelerator were generated for each plan in Eclipse (Varian Medical System, Inc., Palo Alto, CA). Dose-volume statistics were computed and analyzed. A standard constraint set conforming to RTOG 0225 protocol (Radiation Therapy Oncology Group, 2008) (Supplementary Table 1) was used for optimization and evaluation. The aim was to achieve 95% of any PTV at or above the prescription dose, 95-98% of any PTV at or above 95% of the PTV dose, no more than 20% of PTV_7000 at or above 77 Gy (110% of the PTV_7000 dose), and no more than 5% of any PTV_7000 at or above 80.5 Gy (115% of the PTV_7000 dose).

The acceptance criteria for OARs used to define planning objectives were based on QUANTEC-2010 (Quantitative analysis of normal tissue effects in the clinic, 2010) (Supplementary Table 2). The analysis included the mean and maximum dose and a set of appropriate values for V_X_ (percentage volume receiving less/more than X Gy) and D_Y_ (dose received by Y volume). The main considerations in planning the dose are the structures to target and dose-volume parameters, to ensure that the dose limits of crucial structures are not exceeded whilst maintaining sufficient dose coverage.

### Statistical analysis

Both radiation dose and the proportion of doses that exceeded tolerance were calculated for each group. Logistic regression models were applied to quantify the effect of potential risk factors of the radiation doses to OARs. Odds ratios (OR) were calculated to assess the risk of radiation doses for patients from a specific subgroup, relative to the reference. A level of *P* < 0.05 was considered to indicate a statistically significant difference. All analyzes were performed using R3.1.2.

## CONCLUSION

In patients presenting with NPC, the radiation dose to OARs increased significantly with increasing GTV. GTV may be a useful prognostic factor for the radiation dose to OARs around the nasopharynx. Based on the patterns of radiation doses to OARs, it may help us to further reduce subsequent complications by improving the efficiency of plan optimization and evaluation. However, it should be noted that the study did not include the health-related quality of life (HR-QOL) of NPC patients with cancer-free survival after treatment. The median follow-up time for all patients was only 9.8 mouths, and thus lacks analysis of late toxicity. As a result, the relationship between radiation dose of OARs and HR-QOL requires further study.

## SUPPLEMENTARY TABLES


