# Supplementary material for: A prospective study on radiation doses to organs at risk (OARs) during intensity-modulated radiotherapy for nasopharyngeal carcinoma patients
Source: Oncotarget. 2016 Mar 1;7(16):21742–52. doi: 10.18632/oncotarget.7826 (PMC5008319; doi:10.18632/oncotarget.7826)
Supplement: Supplementary file 1 [file oncotarget-07-21742-s001.pdf]

SUPPLEMENTARY TABLES

Supplementary Table 1: Dose constraints used in treatment planning

Structure	Dose volume metrics	Constraint
PTV_7000	V95%¶	≥95-98%
	V100%[#]	≥95%
	V110%[†]	≤20%
	V115%[‖]	≤5%
PTV_6000	V95%¶	≥95-98%
	V100%[#]	≥95%
PTV_5400	V95%¶	≥95-98%
	V100%[#]	≥95%

Abbreviations: PTV: planning target volume;
¶: Percentage dose covering 95% of the PTV
#: Percentage dose covering 100% of the PTV
†: Percentage volume that received > 110% of the Rx (prescribed dose)
||: Percentage volume that received > 115% of the Rx (prescribed dose)

Supplementary Table 2: Dose constraints used for OARs evaluation

Priority	Structure	Dose volume metrics	Constraint
Priority 1: Critical OARs	SpinalCord_PRV	D1[‡]	≤5000cGy
	BrainStem_PRV	D1	≤6000cGy
	OpticNerves_L PRV	D1	≤6000cGy
	OpticNerves_R PRV	D1	≤6000cGy
	Chiasm	Max	≤5400cGy
	Chiasm_PRV	D1	≤6000cGy
	TemporalLobe_L	Max	<6000cGy
	TemporalLobe_L. PRV	D1	<6500cGy
	TemporalLobe_R	Max	<6000cGy
	TemporalLobe_L. PRV	D1	<6500cGy
Priority 2: Intermediate-Risk OARs	Pituitary	Max	<6000cGy
	Mandible_L	V50[®]	<30%
	Mandible_R	V50	<30%
	TM Joint_L	Max	<7000cGy
	TM joint_R	Max	<7000cGy
	Lens_L	Max	<600cGy
	Lens_R	Max	<600cGy
	Eye_L	Mean	<3500cGy
	Eye_R	Mean	<3500cGy
Priority 3: Low-Risk OARs	Parotid_L	Mean	< 26 00cGy
	Parotid_R	Mean	< 2600cGy
	Cochlea_L	Mean	<4500cGy
	Cochlea_R	Mean	<4500cGy
	IAC_L	Mean	<4500cGy
	IAC_R	Mean	<4500cGy
	VestibulSemi_L	Mean	<4500cGy
	VestibulSemi_R	Mean	<4500cGy
	Cochlea_L	Mean	<5000cGy
	Cochlea_R	Mean	<5000Gy
	TympanicCavity_L	Mean	<3400cGy
	TympanicCavity_R	Mean	<3400cGy
	Mastoid_L	Mean	<3000cGy
	Mastoid_R	Mean	<3000cGy
	OralCavity	D1%	<7000cGy

Abbreviations: PRV, planning risk volume; L, light; R, right;
‡: The dose received by 1% of the volume.
®: The percentage volume that received > 50 Gy.

Supplementary Table 3: Mean (± SD) of doses for PTVs based on GTV for the 148 patients

Target	Dose metrics	Group 1	Group 2	Group 3	Group 4	P-value
**PTV_7000**						
	V95 (%)¶	99.42±0.11	99.98±0.04	99.82±0.23	98.72±0.61	0.752
	V100 (%) ®	99.13±0.83	98.84±1.02	97.66±1.77	98.44±1.41	0.678
	V110 (%) †	0.093±0.151	0.069±0.128	0.058±0.083	0.518±0.381	0.135
	V115 (%) &	0	0	0	0	-
**PTV_6000**						
	V95 (%)	99.44±0.35	99.90±0.26	99.94±0.09	99.71±0.25	0.624
	V100 (%)	99.24±0.89	99.35±0.98	99.29±0.41	99.38±0.51	0.541
**PTV_5400**						
	V95 (%)	99.56±0.10	99.47±0.04	99.68±0.12	99.58±0.05	0.489
	V100 (%)	98.66±0.72	99.03±0.72	99.32±0.31	99.37±0.64	0.396

Abbreviations: Group 1: GTV < 20 cm^3; Group 2: 20 ≤ GTV < 40 cm^3; Group 3: 40<GTV < 60 cm^3; Group 4: GTV > 60 cm^3.
¶: Percentage volume covering 95% of the Rx (prescribed dose) ®: Percentage volume covering 100% of the Rx (prescribed dose) †: Percentage volume that received >110% of the Rx (prescribed dose) &: Percentage volume that received >115% of the Rx (prescribed dose)
